# Drug Repositioning through Systematic Mining of Gene Coexpression Networks in Cancer

**DOI:** 10.1371/journal.pone.0165059

**Published:** 2016-11-08

**Authors:** Alexander E. Ivliev, Peter A. C. ‘t Hoen, Dmitrii Borisevich, Yuri Nikolsky, Marina G. Sergeeva

**Affiliations:** 1 A.N. Belozersky Institute of Physico-Chemical Biology, Moscow State University, Moscow, Russia; 2 IP & Science, Thomson Reuters, Boston, Massachusetts, United States of America; 3 Department of Human Genetics, Leiden University Medical Center, Leiden, The Netherlands; 4 Faculty of Bioengineering and Bioinformatics, Lomonosov Moscow State University, Moscow, Russia; 5 Institute for General Genetics, Moscow, Russia; 6 George Mason University, Fairfax, VA, United States of America; 7 Prosapia Genetics, Solana Beach, California, United States of America; Semmelweis University, HUNGARY

## Abstract

Gene coexpression network analysis is a powerful “data-driven” approach essential for understanding cancer biology and mechanisms of tumor development. Yet, despite the completion of thousands of studies on cancer gene expression, there have been few attempts to normalize and integrate co-expression data from scattered sources in a concise “meta-analysis” framework. We generated such a resource by exploring gene coexpression networks in 82 microarray datasets from 9 major human cancer types. The analysis was conducted using an elaborate weighted gene coexpression network (WGCNA) methodology and identified over 3,000 robust gene coexpression modules. The modules covered a range of known tumor features, such as proliferation, extracellular matrix remodeling, hypoxia, inflammation, angiogenesis, tumor differentiation programs, specific signaling pathways, genomic alterations, and biomarkers of individual tumor subtypes. To prioritize genes with respect to those tumor features, we ranked genes within each module by connectivity, leading to identification of module-specific functionally prominent hub genes. To showcase the utility of this network information, we positioned known cancer drug targets within the coexpression networks and predicted that Anakinra, an anti-rheumatoid therapeutic agent, may be promising for development in colorectal cancer. We offer a comprehensive, normalized and well documented collection of >3000 gene coexpression modules in a variety of cancers as a rich data resource to facilitate further progress in cancer research.

## Introduction

Cancer is a multifactorial disease driven by “hallmarks”, such as proliferation, invasion, angiogenesis, metastasis development and other contributing factors [1]. Understanding the molecular basis for these oncogenic hallmarks is crucial for the development of cancer therapies. With the advent of high-throughput expression profiling technologies, these mechanisms are being increasingly dissected through analysis of cancer transcriptome, with applications in patient stratification, drug resistance and therapy development.

An established strategy in cancer transcriptomics is meta-analysis of gene expression datasets [2,3]. Unlike the approach focused on a single study, meta-analysis considers multiple independent datasets jointly. This increases robustness of results and allows uncovering biological connections that may not be evident from the individual datasets [2,4]. Meta-analysis has long been widely used in oncology. For example, in a large expression compendium, Rhodes *et al* identified the angiotensin receptor to be consistently overexpressed in a specific breast cancer patient subpopulation [5]. This suggested a possibility to treat these cancers using angiotensin receptor antagonists such as losartan–a non-obvious antihypertensive medication [5]. As gene expression data continue accumulating in public repositories, meta-analysis becomes increasingly powerful as a strategy to address key problems in cancer research [6,7].

In both single and meta-analyzed studies, a question widely asked by researchers is which genes are differentially expressed between experimental conditions. While powerful, differential expression analysis focuses on how transcriptome is connected to external sample characteristics but ignores the intrinsic biological structure of a dataset. It also assumes that genes change expression levels independently of each other, which is clearly not the case in many biological systems, since biological complexity originates from functional interactions between genes and proteins.

A complementary approach–gene coexpression networks–aims to explore such gene-gene relationships [8,9]. It does so by assessing gene-gene correlations within a transcriptomic dataset. As has been shown in a number of studies, genes co-expressed in a cell or a tissue typically do not exhibit such an organized, coordinated behavior by coincidence but are rather driven by shared higher-level processes that functionally connect them [10,11]. A transcriptome with thousands of genes is thereby delineated into a handful of distinct coexpression modules corresponding to meaningful processes and pathways. This highlights gene involvement in critical functions within cancer cells.

Weighted Gene Coexpression Network Analysis (WGCNA) is an advanced and thoroughly corroborated methodology for reconstructing coexpression networks [12,13]. Unlike basic coexpression approaches that examine each gene pair separately from the rest of the transcriptome, WGCNA employs a more integrative measure of gene coexpression, known as topological overlap. Topological overlap defines gene relatedness by considering not only the correlation of two genes with each other, but also whether these genes are correlated with similar sets of genes across the entire transcriptome [13]. This more holistic coexpression measure downplays spurious correlations and leads to robust gene modules–eventually resulting in a clearer view of the examined transcriptome [14,15].

Previous studies applied WGCNA to patient stratification, gene function prediction and understanding biology of drug targets [16–18]. Here, we aimed to systematically explore functional transcriptome organization at high resolution in common cancers using WGCNA and make this information available to the scientific community. For biologists, high-resolution coexpression information would offer insights into key disease processes in cancer cells. For computationally advanced bioiformaticians, such data would be valuable as a resource for data mining and for integrating into broader multi-omics computational workflows.

Towards that end, we reconstruct gene coexpression networks in 82 datasets from 9 major human cancer types. In these networks, we identify numerous gene coexpression modules and associate them with pathological processes in cancer cells. We further identify central (“hub”) genes in each module that potentially play key roles in respective cancer cell functions. To showcase the utility of this information, we apply these data to predict a potential novel indication for Anakinra–an antirheumatoid drug with prior evidence of anti-cancer effects. The compendium of high-resolution cancer gene coexpression modules is available at http://wgcna-modules.appspot.com/.

## Materials and Methods

### Data Preprocessing

Before data preprocessing, we first searched GEO for Affymetrix-based microarray gene expression datasets as described in the Results section. After identifying and downloading the datasets, we removed normal samples (if any) from the datasets. All datasets were next normalized using a common procedure for consistency (one dataset at a time). The normalization was performed using custom gene-level Chip Definition Files, where non-specific and mis-targeted probes are masked (http://masker.nci.nih.gov/ev/). Since the custom CDFs are already defined at the level of Entrez IDs, we did not need to perform any probeset-to-gene summarization. Each dataset was normalized using MAS5 algorithm followed by quantile normalization, similar to the previous studies [11,16]. To remove outlier samples, Pearson correlations were computed for each sample against every other sample within a dataset, and samples with unusually low average correlations were removed (4 square deviations below the overall mean in the dataset) [11,19]. The pre-processing resulted in datasets with 18,835 and 12,287 genes for the U133Plus 2.0 and U133A microarray platforms, respectively.

### Weighted Gene Coexpression Network Analysis

Coexpression networks were constructed independently in each dataset. In line with the WGCNA workflow, we first calculated Pearson correlations between all genes present in a given dataset, resulting in an all-against-all gene correlations matrix. The correlations were next raised to a power β to penalize weak correlations while preserving stronger ones. The procedure is known as “soft thresholding” of a network–in contrast to “hard thresholding” where a network is filtered using an abrupt cutoff [12,13]. The β value for the soft thresholding was chosen in each dataset independently from the 7–15 range using the “scale free topology criterion” proposed by Zhang and Horvath [12]. We used the “signed” version of soft thresholding. The procedure resulted in a weighted network also known as an adjacency matrix, where adjacency = correlation^β^.

As a next step, the adjacency matrix was transformed into a network of Topological Overlaps (TO). TO quantifies coexpression relationships considering each pair of genes in relation to all the other genes in the network. For a pair of genes *i* and *j*, *TO* was computed as originally described:

$$\frac{\sum_{u}a_{iu}a_{ju}+a_{ij}}{\min\{\sum_{u}a_{iu},\sum_{u}a_{ju}\}-a_{ij}+1}$$

where *a* denotes adjacency between two genes defined by the subscripts [12,13]. TO eliminates spurious correlations and reinforces consistent patterns of network connections to allow for more robust transcriptome exploration [12,13].

After constructing the TO network, we hierarchically clustered genes using 1 –TO as a distance measure. This resulted in a cluster dendrogram, with branches corresponding to gene coexpression modules [20]. The module definition in the dendrograms was performed using the dynamicTreeCut algorithm with the following parameters: mode–“tree”, deep split–“false”, maximum cut height– 0.995, minimal module size– 15 genes [20].

The resulting modules are groups of genes with consistently correlated expression profiles across samples within a given dataset. Each module can be characterized by a dominating expression trend, also known as a module eigengene (ME). An eigengene summarizes the expression profiles of all genes in a given module as a single meta-profile, which serves as a condensed representation of the module. Eigengenes were calculated as a first principal component of the expression profiles of all genes in a module–a standard approach in WGCNA [13]. In each dataset, we merged modules with highly correlated eigengenes (Pearson correlation > 0.8) [11]. We also removed genes weakly correlated with the module eigengene (Pearson correlation < 0.3).

### Robustness Analysis

A subset of modules may correspond to rare biological events which are less interesting for further analysis. A typical example is a genomic alteration found in a single patient or a small patient subpopulation in a cancer type [21]. To filter out such modules that only correspond to rare biological events, we assessed the robustness of the modules using the module density measure.

We defined module density as average correlation between expression profiles of all genes in a given module [11,13]. An abrupt decrease in density of a module upon removal of only few samples from a dataset indicates that the module is highly dependent on the removed samples and largely disintegrates once these samples are removed from the dataset. This suggests the module to be driven by a rare event, e.g. a highly uncommon genomic deletion or amplification found only in those samples.

To perform the filtering, we calculated two density values per coexpression module: 1) density across all samples in the dataset; 2) density after excluding the 1% of the samples with highest value of the module eigengene. Modules with an over two-fold decrease in density upon the removal of these samples were excluded from further analysis.

### Gene Connectivity within Modules

Genes in a module can be prioritized by their connectivity with the rest of the genes in the module. Highly connected genes represent central (“hub”) genes in the module, while lowly connected ones can be interpreted as peripheral genes. We used two measures to quantify this gene quality. The first measure, intramodular connectivity, was defined as average topological overlap between a gene of interest and all the other genes in its module of residence[12]. For convenience of representation, we scaled these connectivity values to the [0;1] interval. We also calculated module membership (kME)–a complementary measure of gene association strength with coexpression modules. In contrast to intramodular connectivity, kME can be calculated for a gene with respect to any module–not only it’s module of residence. The measures is defined as Pearson correlation between the gene expression profile and the eigengene of a module [13].

### Module Functional Annotation

After identifying the modules, we annotated them functionally. This was done by testing each module for enrichment in 3 types of gene sets: (1) Gene Ontology biological processes (3,941 GO terms ranging from 10 to 1,000 genes in size); (2) chromosomal cytobands (1q1, 1q11, 1q12, etc.); and (3) tissue-specific gene sets corresponding to 36 normal human tissues [22]. The enrichments were calculated using hypergeometric test with a Benjamini-Hochberg multiple testing correction.

### Drug Repositioning Analysis

In the analysis of the modules in the context of extracellular matrix therapies, we first searched our compendium for the modules associated with cell adhesion to extracellular matrix. To that end, in each dataset, we tested each module for an enrichment in the “cell adhesion” GO term and selected a module that was enriched stronger than any other module in that dataset (provided also that P < 10^−5^). The module (further referred to as extracellular matrix module–ECM module) was then tested for presence of therapeutic targets of cancer drugs and other drugs from DrugBank (http://www.drugbank.ca/, v. 4.0). In this analysis, we first explored target occurrence in the ECM module across all datasets, taking into account only targets found *within* the ECM module (S1 Table, spreadsheet #1). Second, a more comprehensive analysis was performed for the *IL1R1* target, where we tested this target’s strength of association with the ECM module regardless of whether *IL1R1* was found within this module or outside of it (S1 Table, spreadsheet #2). The association was measured as Pearson correlation between the expression profile of *IL1R1* and eigengene of the ECM module (the kME measure). The different cancer types were finally compared in terms of the IL1R1-ECM association strength to identify cancers where *IL1R1* is promising as a target and may impact extracellular matrix biology that facilitates tumor growth.

## Results

### Mining Gene Coexpression Modules across Multiple Cancer Types

As a starting point for gene coexpression network analysis, we selected datasets in Gene Expression Omnibus (GEO) using the following criteria: (1) dataset contains at least 30 samples corresponding to tumors with a shared anatomical location (breast, lung, gastric cancer, etc); (2) microarray platform–Affymetrix U133A or U133 Plus 2.0; (3) raw data available. We focused on cancer types with at least 3 available datasets. For cancer types with over 20 suitable datasets, we took the 20 largest studies. We also ensured that datasets have no sample overlap. The search resulted in 82 datasets corresponding to 9 major cancer types: breast, colon, lung, ovarian, kidney, gastric and prostate cancers, as well as glioma and melanoma. While GEO includes all datasets in pre-processed formats, the preprocessing methodologies are heterogeneous, potentially leading to excessive technical variation. We therefore downloaded the datasets as raw CEL files and preprocessed them independently using a consistent procedure identical for all the datasets (see Methods for details and S1 Table for a datasets description).

The data collection is briefly characterized in Table 1. Columns in this table include cancer type names, numbers of available datasets and dataset sizes. The table also shows how many modules were ultimately identified in the datasets. Each cancer type was represented by 3 to 20 datasets, with lowest for gastric and highest for breast, colon and lung cancer types (Table 1). The variation is related to data availability in the GEO repository, potentially reflecting cancer epidemiology or activity of scientific research. By total sample counts, data were most abundant for breast, colon and brain cancers, with over 1,000 samples for each of these cancer types (Table 1).

**Table 1:** Overview of datasets and transcriptional modules.

Cancer type	Number of datasets	Total number of samples	Total number of modules^1^	Median number of samples (per dataset)	Median number of modules (per dataset)^1^
All types	82	8422	3,398	80	38
Breast	20	2899	726	118	32
Colon	14	1441	686	90	52
Gastric	3	281	149	43	57
Glioma	11	1018	425	74	39
Kidney	6	443	239	65	37
Lung	12	946	539	66	44
Melanoma	5	271	227	63	47
Ovarian	7	845	282	99	32
Prostate	4	278	125	65	29

1 –after exclusion of modules associated with rare biological events (Methods)

**Fig 1:**
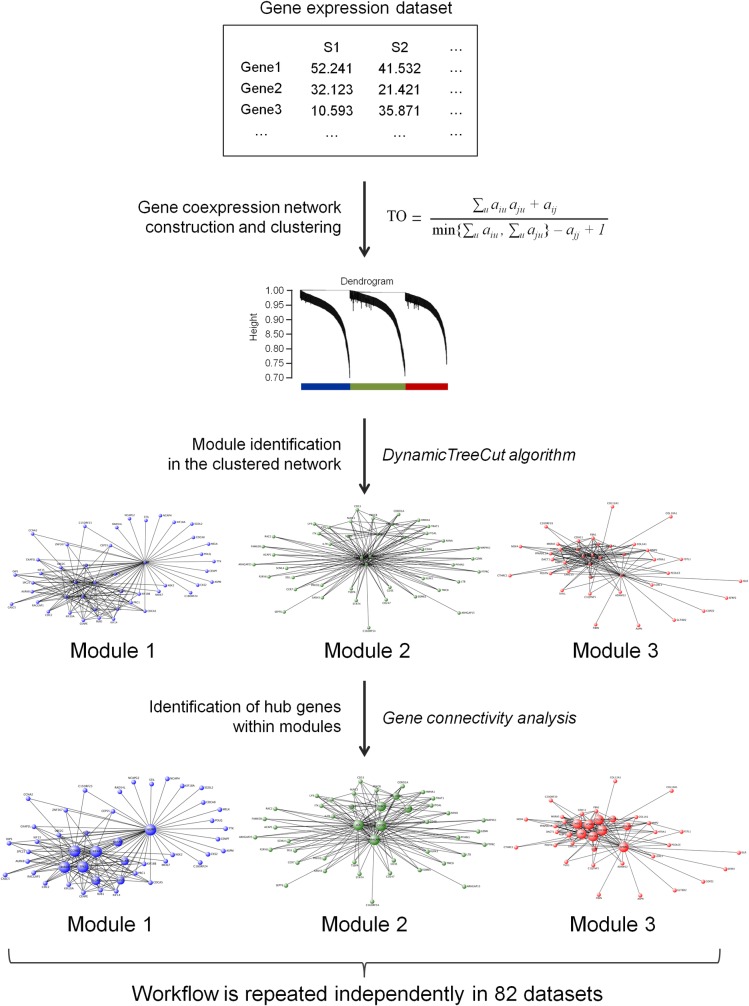
Workflow overview. In each dataset, the following workflow was applied. 1. The dataset was used as a starting point to construct a gene coexpression network based on Topological Overlap between genes. TO determines similarity between gene expression profiles taking into account a systems level context. The network was next hierarchically clustered, resulting in a cluster dendrogram. 2. Using DynamicTreeCut algorithm, branches were identified in the dendrogram, leading to identification of gene coexpression modules. 3. Genes in each module were further prioritized by intramodular connectivity, providing a distinction between lowly and highly connected genes. The entire workflow was repeated independently for 82 datasets, resulting in a set of gene coexpression modules in each of them.

To systematically explore the transcriptome organization in each of these 9 cancer types, we reconstructed gene coexpression networks independently in the 82 datasets using the WGCNA algorithm (see Fig 1 for an overview). In a given dataset, the analysis started out by calculating gene-gene Pearson correlations. The Pearson correlations were next weighted and transformed into Topological Overlap (see Methods for details). Based on the Topological Overlap networks, genes were finally clustered hierarchically, resulting in a dendrogram with branches corresponding to modules of coexpressed genes in a particular dataset. The analysis identified 3,398 gene coexpression modules in total across the datasets (Table 1). To the best of our knowledge, these data represent the most comprehensive collection of gene coexpression modules generated in oncology to date.

### Gene Coexpression Modules Overview

As follows from Table 1, each dataset included a number of gene coexpression modules. The modules ranged from 13 to over 2,000 genes in size (with a median of ~100 genes per module). Names of the individual genes residing in each module are listed in S2 Table. This table also shows gene connectivity values that characterize how strongly each gene is associated with its module of residence (see below). Similar data with further details are also provided dynamically at http://wgcna-modules.appspot.com/.

Which questions are being addressed by these data? For a gene of interest, this information uncovers transcriptional modules that implicate this gene in the various cancer types. Taking into account module associations with biological processes (S3 Table), genes can be further connected to critical functions in cancer cells. From a complementary process-centric perspective, this also characterizes the biological processes, suggesting gene players involved in these functions and potentially contributing to cancer growth.

### Module Biological Functions

As previously discussed, gene modules are typically associated with biological processes underlying the coexpression in them. To associate the modules with biological processes and functions, we therefore used functional enrichment analysis–a widely used bioinformatics technique [23]. Functions significantly overrepresented in a module are likely to drive the coexpression of the genes [9,23]. We separately annotated modules through this approach with respect to (1) biological processes, (2) chromosomal locations and (3) tissue specificity markers (S3 Table; see Methods for details).

**Fig 2:**
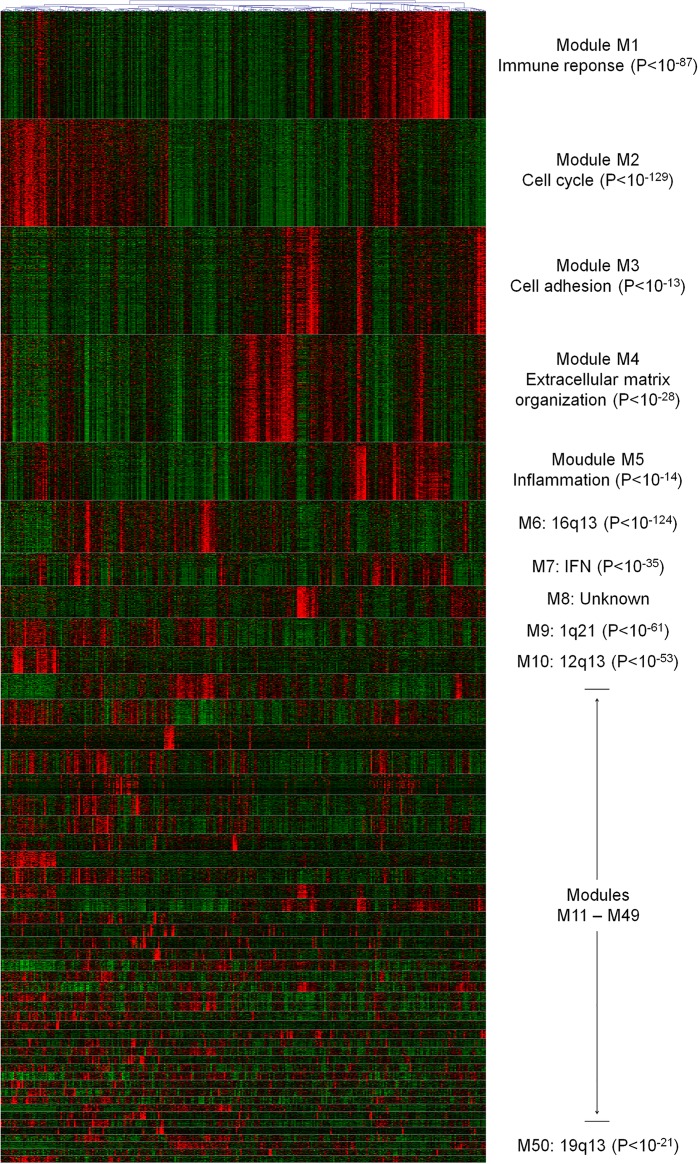
Modules in a GSE20865 breast cancer dataset. GSE20865 was the largest breast cancer dataset analyzed here and includes 327 patients. The coexpression network identified 50 modules in this dataset. This heatmap displays expression patterns of genes in each module: with genes in rows and patients in columns. The modules larger than 250 genes (M1—M4) are represented by only the top 250 highly connected genes (to facilitate compact visualization). For selected modules, key biological functions are specified, with corresponding enrichment P-values.

Fig 2 illustrates module functions for a specific cancer dataset. This dataset (the largest in our study) included approximately 300 breast cancer patients and resulted in 50 modules at the network analysis step. According to the enrichment analysis, the modules were associated with various biological themes (S3 Table, dataset #1): 1) classical cancer-related processes, e.g. immunity (M1), proliferation (M2) and extracellular matrix remodeling (M4); 2) signaling pathways, e.g. interferon signaling (M7) and steroid hormones (M33); 3) breast cancer subtypes, e.g. basal (M2), normal-like (M21) and possibly neuroendocrine (M13) subtypes; 4) genomic alterations: 1q42, 8q24, 11q13, 17q25, 19p13, 22q13, etc (approximately 30 modules in this category). Finally, some modules demonstrated no functional enrichments.

**Fig 3:**
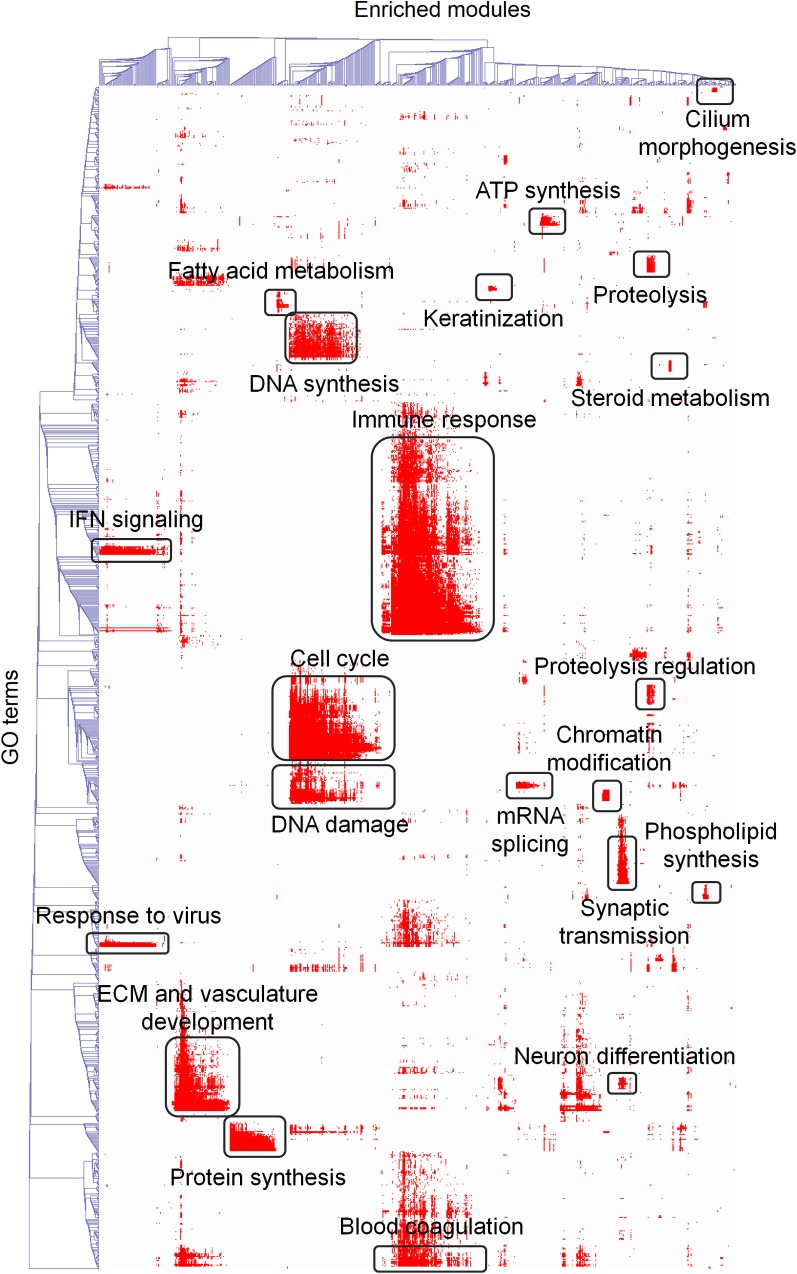
Cross-dataset high level functional landscape. This heatmap displays associations between gene coexpression modules and biological processes across all the datasets. Color denotes enrichment of a given module with a biological process: hypergeometric log p-value after Benjamini-Hochberg adjustment. Cluster height reflects how many interrelated processes are associated with the given module set: the higher a cluster–the broader is the module-associated functional theme. Cluster width reflects how many modules are sharing this function: the wider a cluster–the more frequently this function is found in the GEO datasets. For major clusters, key biological themes are subscribed. The heatmap includes 1,240 biological processes and 668 modules, which were selected as follows. A GO process was included if it’s associated 3 or more coexpression modules (P < 0.001). A module was included if it’s enriched with 3 or more biological process terms (P < 0.001).

From a broader perspective–at a bird’s eye view across all datasets–frequent functions included cell cycle, immunity, angiogenesis, mitochondrial respiration/glycolysis (the alternative tumor metabolism modes), hypoxia response, fatty acid oxidation, steroid biosynthesis, protein glycosylation and others (Fig 3). In this heatmap, rows correspond to biological processes, whereas columns–to associated modules of coexpressed genes. Large colored areas reveal module assemblies associated with process sets. For example, “cell cycle” (a coherent process group consisting of “S phase”, “mitosis”, etc) is a prevalent function that, unsurprisingly, has a matching module in all the cancer datasets. Interestingly, “cell cycle” was also closely related to (but distinct from) “DNA synthesis” and “DNA damage response” (Fig 3). This suggests that respective processes are highly correlated but distinct in cancer cells. Overall, the functional landscape described here (Fig 3) confirms that the modular transcriptome organization captures diverse aspects of cancer as a disease.

**Fig 4:**
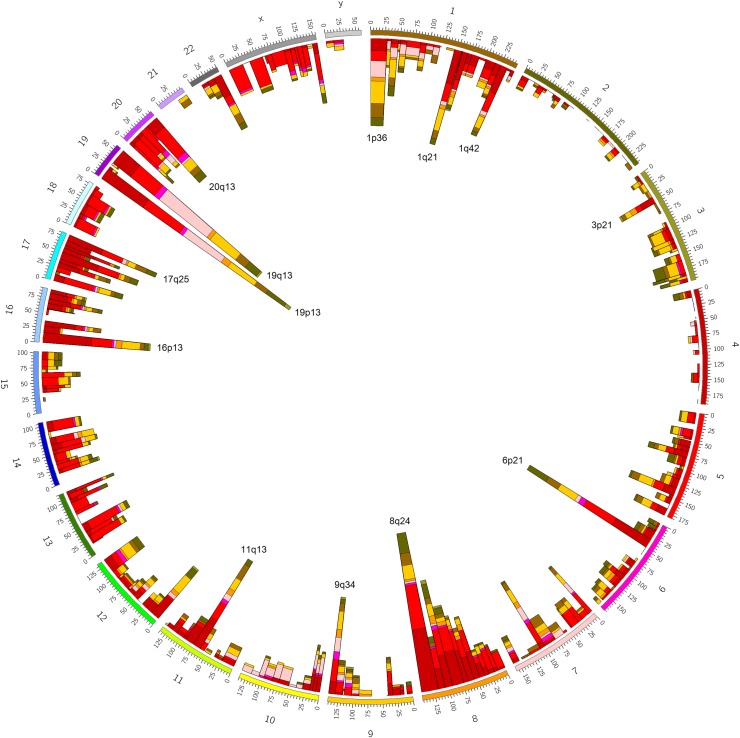
Module enrichments with chromosomal cytobands. Numbers on the outer side of the circle are chromosomes. Coordinates within each chromosome are genomic coordinates. Bar height on the inner side of the circle is proportional to number of modules from a given cancer type enriched with a respective cytoband at P < 10^−3^. Dark red: breast cancer; red: colon cancer; magenta: glioma; pink: lung cancer; orange: ovarian cancer; yellow: prostate cancer; brown: kidney cancer; dark green: gastric cancer; light green: melanoma. Visualization was produced using Circos software [50].

Besides biological processes, the modules were also enriched with tissue-specific gene markers and chromosomal locations (S3 Table, columns E and F). Indeed, over 800 of the 3,398 modules were strongly enriched in chromosomal locations at P < 10^−15^ (see Fig 4 for an overview). This potentially reflects DNA copy number alterations (CNAs), consistent with genomic instability representing a key feature of cancer cells [24]. In terms of tissue-specific gene markers, the tissue enrichments tended to be associated with modules from “correct” cancer types. For example, brain marker enrichments were strongest in glioma, whereas kidney markers–in kidney cancer datasets, etc (S3 Table column F). The tissue-marker modules may correspond to normal cells embedded in the tumors–but also, more intriguingly, to genetic programs of differentiation in cancer cells themselves [25,26].

As a final observation, the enrichment analysis revealed modules that were not enriched with any function, tissue or chromosomal location. This suggests that some of the cancer transcriptional variation may relate to yet unknown cancer functionality. While it remains to be determined which of these modules are related to cancer pathology, some of them might represent an interesting subject for further research.

### Identification of Hub Genes within Coexpression Modules

Highly connected (hub) genes in molecular networks are thought to play prominent roles in various biological systems [27,28]. Highly connected genes within modules may be critical for module-specific biological mechanisms [28,29]. Previous studies also reported hub genes from both coexpression and protein interaction networks as more essential for organism survival and more evolutionarily conserved than average genes in respective organisms [27,28]. In light of these findings, “hubs” are of interest from a practical perspective, such as drug target identification [18] and understanding of disease mechanisms [11,14]

In connection with this, we identified highly connected genes in each coexpression module. The connectivity was measured as a sum of connection strengths between a gene of interest and all other genes in a given module (Methods). This resulted in a prioritization of genes within each coexpression module (S2 Table).

**Fig 5:**
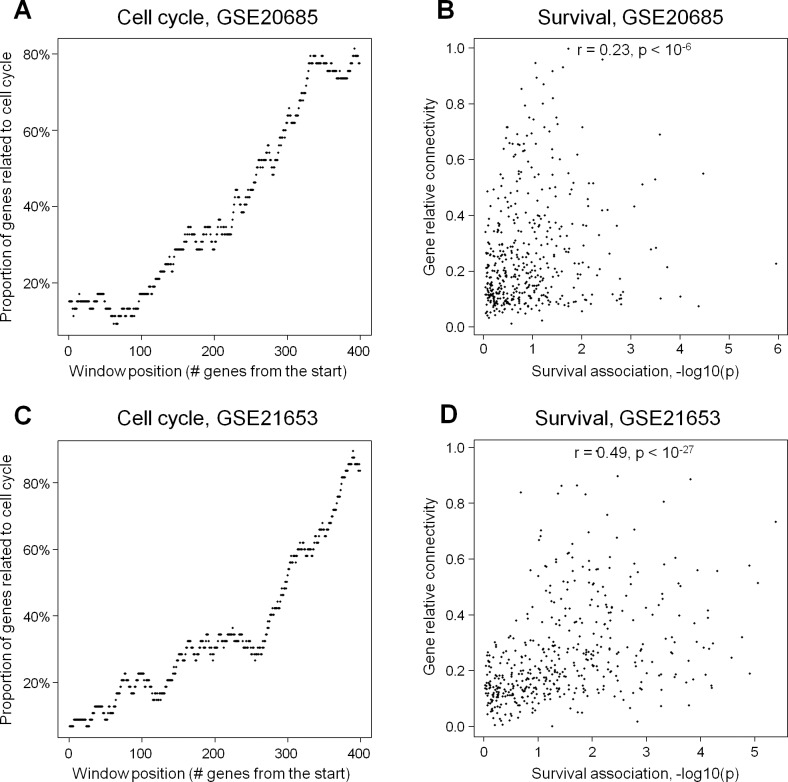
Gene connectivity in the proliferation module: highly connected genes are associated with relevant biology and poor survival prognosis. Figures A and B correspond to the GSE20685 dataset (the largest breast cancer dataset in our study); C and D–to GSE21653 (the second largest dataset). A and C: proportion of genes related to the cell cycle GO process in a 50-gene window sliding from lowly to highly connected genes. B and D: scatter plots for gene connectivity against the power of a gene to predictive survival. The gene predictive power was defined as–log(P) from Cox univariate survival regression. Spearman correlations and statistical significance values are shown.

High connectivity sheds light on gene functions in a module of interest [18]. To test this hypothesis, we examined module #2 (the “cell cycle” module) from the already described Fig 2. Module enriched with the same function has recently gained attention as a predictor of patient survival in multiple independent breast cancer cohorts [3,30]. In our study, a gene’s probability of being related to cell cycle is strikingly correlated with intramodular connectivity of the gene in this module. Indeed, the proportion of genes involved in cell cycle grew steadily from 10–15% among the lowly connected genes, up to 80–90% among the highly connected genes in the module (Fig 5A). Separately, we tested how gene connectivity in the same module was related to patient survival time. The connectivity was found to significantly correlate with gene’s power to predict survival (Fig 5B). Both findings suggest that connectivity is important as a biological characteristic and can be useful for cancer research. This is consistent with previous findings of highly connected genes as relevant to target [18] and biomarker identification [16].

### Drug Repositioning using Gene Coexpression Modules

One of the applications from our molecular networks is the identification of therapeutic targets and drugs [31,32]. As a showcase, we performed an analysis known as drug repositioning. The analysis generates hypotheses on whether a drug can be used outside of its existing approved indications. Drug repositioning is quicker and less risky in clinical trials compared with completely de novo drug development [33]. Here, we sought to predict drugs approved outside of oncology but tentatively promising in cancer, with a focus on extracellular matrix biology–a mechanism actively pursued in cancer drug industry in recent years [34,35].

To that end, we searched and examined an extracellular matrix module (further “ECM” module) that was broadly represented in our datasets. The module was present in 76 out of the 82 datasets and was enriched in extracellular matrix and cell adhesion functions (P < 10^−5^, S4 Table, tab 1, row 4). These functions play major roles in tumor growth and invasion [34,35], making it unsurprising that the module was found in a high proportion of the datasets.

To analyze this module from the drug development perspective, we tested whether it harbors targets of known anti-cancer drugs (S4 Table). As an initial observation, the ECM module did frequently include well-known oncology targets, supporting its relevance to cancer therapy research. As an example, metalloproteinase proteins targeted by marimastat (*MMP1*, *MMP2*, *MMP3* and others) were present in the ECM module in multiple datasets. Other established oncology targets included: *PDGFR* (modulated by Regorafenib and Sunitinib), *FLT1* (Axitinib), *MAP1* and *MAP2* (Estramustine), *ABL1* (Bosutinib), *TEK* (Regorafenib), and several others.

We further searched the module for targets of drugs that are currently not approved for cancer treatment. Such targets in the module included: endothelin receptor *ENDRA* (modulated by Bosentan), integrin *ITGA4* (Natalizumab), integrin *ITGB3* (Eptifibatide), immunoglobulin receptor *MS4A2* (Omalizumab), *AGTR1* (Azilsartan) and several others (S4 Table). The targets’ presence in the module of interest suggests these drugs to potentially impact extracellular matrix biology and serve as possible candidates for further research in this respect.

We further noted an interesting pattern of membership exhibited in the ECM module by interleukin 1 receptor (*IL1R1*). This target is known to be modulated by a therapeutic agent Anakinra currently used to treat rheumatoid arthritis. *IL1R1* was notable for its seemingly strong association with the ECM module in colorectal cancer but not in the other cancer types (S4 Table). To confirm this observation, we examined the correlation between *IL1R1* expression level and eigengene of the entire ECM module in each of the different datasets (Methods). Such gene-to-module correlation determines how strongly a gene is associated with a module of interest, without requiring this gene to be present in the module in every dataset (see Methods, “Gene connectivity” section). We found the IL1R1-ECM correlation to be specifically increased in colorectal cancer (Pearson coefficient 0.74 on average) compared with non-colorectal datasets (0.3 on average, P < 10^−6^ in Mann-Whitney test, Table 2). This suggests that Anakinra’s target *IL1R1* is stronger associated with extracellular matrix biology in colorectal cancer compared with the other cancer types. While further research will be necessary, these results indicate Anakinra as a potential agent to modulate extracellular matrix biology in solid tumors–with a focus of further research on the colorectal cancer type.

**Table 2:** Association of IL1R1 gene with the extracellular matrix module.

Cancer Type	ECM module count^1^	IL1R1-positive ECM module count^2^	Ratio^3^	Correlation^4^
Colon cancer	14	11	0.79	0.74
Gastric cancer	3	1	0.33	0.58
Glioma	8	2	0.25	0.54
Melanoma	4	1	0.25	0.28
Breast cancer	20	3	0.15	0.40
Lung cancer	11	1	0.09	0.30
Kidney cancer	5	0	0.00	0.09
Ovarian cancer	7	0	0.00	0.02
Prostate cancer	4	0	0.00	-0.14

1 –number of datasets from a given cancer type containing the ECM module

2 –number of datasets from a given cancer type containing IL1R1 within the ECM module

3 –ratio between the second and the first columns

4 –Pearson correlation between IL1R1 expression profile and the eigengene of the ECM module. Each value represents an average of the correlations across all ECM module-containing datasets of a given cancer type.

## Discussion

Cancer transcriptome is a complex system, reflecting tumor heterogeneity and various molecular mechanisms of the disease. This complexity poses a challenge and requires systematic approaches for transforming data into knowledge and actionable hypotheses for clinical research. To improve current understanding of the cancer transcriptome, we explored gene networks in 9 major human cancer types using a compendium of publicly available data. The analysis resulted in a large collection of high-resolution robust gene coexpression modules which offer insight in cancer biology.

Our approach differs from previous gene expression meta-analyses in oncology in several respects. First, we use WGCNA methodology which enables robust and sensitive detection of gene coexpression modules even in complex transcriptomes, such as cancer and human brain [11,12,36]. Second, our strategy is purely data-driven–in contrast to knowledge-based and hybrid approaches utilized in some of the previous research [37,38]. As an example, Segal *et al* created an assembly of cancer-related gene modules using a hybrid approach that used a collection of predefined gene sets as a starting point [37]. While knowledge reinforces data interpretation, it also limits one’s ability to discover entirely novel molecular changes–a pitfall circumvented by our approach. Third (and last), our approach is independent of healthy samples. Several previous studies focused on coexpression patterns in cancer as opposed to normal transcriptome [39,40]. While intuitive, such strategy is prone to exclusion of disease-related modules that only superficially resemble normal ones. One such example is modules enriched in normal neuron- and astrocyte-specific genes in brain tumors. These modules (despite the mentioned enrichments) in fact correspond to proneural and proastrocytic patterns of tumor cell differentiation (important diagnostic criteria) rather than to normal neurons or astrocytes themselves [16,41,42]. Taken together, the unbiased WGCNA strategy in the present study circumvents some of the limitations from prior research.

From a computational perspective, it should also be noted that coexpression studies often focus on only a subset of genes from the entire transcriptome. This is due to the fact that memory requirements grow quadratically with the number of genes, making large networks technically challenging to analyze. Here, we analyze gene coexpression networks at the entire transcriptome scale–without pre-filtering–to avoid any loss of useful information.

In this study, we used Affymetrix microarrays as a basis for the analysis. In recent years, RNA-seq became widely used as a tool for expression profiling. RNA-seq offers advantages over microarrays in accurate quantification of low abundance genes and novel transcript discovery. Meanwhile, the two technologies are in a reasonably good agreement with respect to the rest of the genes and often lead to common biological conclusions [43,44]. Given the wealth of data accumulated using microarrays over the past decade, these data will remain of value for research, while being complemented by the growing amount of RNA-seq data.

The data generated here may serve various applications in oncology. The modules predict gene functions by linking genes to their coexpression partners with the already known oncology roles [9]. At a level of biological processes, the modules also provide molecular portraits of key cancer functions in terms of the transcriptome. Gene connectivity provides further resolution and allows for comparing genes by strength of their association with key processes in the disease.

For convenience, the data are provided in two formats: a user friendly interface (http://wgcna-modules.appspot.com/) and a bulk download gene connectivity data matrix (same location). The second option is more efficient for computational use such as, for instance, predicting drug targets or cancer drivers through machine learning analysis [45].

One of the possible uses of network information relates to various forms of drug research. The analysis performed in our study suggests that a multi-dataset design offers advantages over the traditional single-dataset setting. Thus, a target of Anakinra (*IL1R1*) was associated with the module of extracellular matrix biology distinctly in the different cancer types. This observation would not be evident in case the study included only a single cancer dataset or multiple datasets from a single type of cancer. Anakinra is an anti-rheumatoid agent which acts by binding the receptor of interleukin-1 in the plasma membrane and prevents interleukin-1 from fulfilling its normal physiological functions [46]. Besides its use in rheumatoid arthritis, recent data suggest Anakinra to offer promising anticancer effects. Anakinra influences tumor microenvironment and extracellular matrix processes in experimental models of melanoma [47], lung cancer [48] and several other cancers [49]. Our analysis suggests that Anakinra may have unique effects in colorectal cancer where its target is associated with the extracellular matrix module stronger than in the other examined cancer types. While therapeutic potential of this agent requires further investigation, this hypothesis illustrates an advantage of meta-analysis compared with smaller-scale approaches focused on a particular cancer type.

In conclusion, gene coexpression networks in this study provide insight in cancer biology as a complex and multifaceted disease. These data facilitate hypothesis generation and will serve as a useful resource for the community to further support oncology research.

## Supporting Information

S1 Table S1 Table characterizes datasets included in the study: platforms, number of samples, authors, etc. (XLSX)

S2 Table S2 Table contains 3,398 gene coexpression modules in the format of gene lists, including gene connectivity values. Similar data in the matrix format for programmatic use are available on the web at http://wgcna-modules.appspot.com/.
(XLSX)

S3 Table S3 Table provides functional annotation to gene coexpression modules. (XLSX)

S4 Table S4 Table contains supporting information for the extracellular matrix module case study. (XLSX)
